# A Complete Workup of Recurrent Syncope Caused by Significant Left Main Coronary Artery Stenosis

**DOI:** 10.7759/cureus.25909

**Published:** 2022-06-13

**Authors:** Seyed Zaidi, Rafsan Ahmed, Ahmad Jallad

**Affiliations:** 1 Cardiology, State University of New York Downstate Health Sciences University, Brooklyn, USA; 2 Internal Medicine, State University of New York Downstate Health Sciences University, Brooklyn, USA

**Keywords:** percutaneous coronary intervention, coronary angiogram, ischemic cardiomyopathy, coronary artery disease, atrial tachycardia, ventricular tachycardia, left main coronary artery stenosis, recurrent syncope

## Abstract

Syncope is usually caused by cerebral hypoperfusion. Differentials to consider during the workup of syncope includes vasovagal, orthostatic, drug-induced, arrhythmia, structural heart disease, and ischemic cardiomyopathy.

An 81-year-old African American man with recurrent witnessed syncopal events and newly diagnosed heart failure underwent extensive cardiac workup including electrocardiograms (EKG), echocardiogram, Holter monitor, electrophysiology (EP) study, and coronary angiogram. The workup revealed ischemic ventricular tachycardia in the setting of significant coronary artery disease including 80% distal left main disease. The patient underwent a coronary artery bypass graft (CABG) with subsequent resolution of further syncopal events. The patient was successfully discharged with guideline-directed medical therapy for heart failure with reduced ejection fraction (HFrEF) and coronary artery disease (CAD).

It is very rare for ischemic cardiomyopathy to present as syncope; however, it is not unheard of. Extensive transmural ischemia could lead to ventricular arrhythmias, a known cause of syncope. This rare presentation serves as a reminder to consider ischemic heart disease in the evaluation of syncope.

## Introduction

Syncope as an initial presentation of ischemic heart disease is exceedingly rare [1]. Cardiac diseases account for 8.3-23% of all causes of syncope [2-4]. Left main coronary artery (LMCA) stenosis presenting as syncope has been reported [5,6]. Here, we present a case of recurrent syncope attributed to LMCA stenosis.

## Case presentation

Our patient is an 81-year-old male with a past medical history of hypertension, benign prostatic hyperplasia (BPH), and iron deficiency anemia attributed to chronic hemorrhoids. He admitted experiencing pre-syncopal and syncopal events for two months which began with a sudden sensation of lightheadedness associated with diaphoresis; subsequently, he was minimally or completely unresponsive witnessed by his wife, lasting 10-15 s, without any convulsive behavior, post-ictal state, or loss of bowel or urinary continence. Additionally, he denied any headache, nausea, vomiting, chest pain, or shortness of breath during these episodes. His baseline hemoglobin was 8.8 g/dL; nevertheless, his recent colonoscopy last year was unremarkable. His medications included amlodipine 5 mg daily, Ferrous sulfate 350 mg daily, docusate calcium 240 mg daily, omeprazole 20 mg daily, and tamsulosin 0.4 mg daily. 

The patient had multiple visits to the emergency department (ED) including two hospital admissions for syncopal events. His vitals were notable only for hypertension with systolic blood pressure ~140 s and diastolic of ~80 s. Orthostatic vitals remained negative and physical exams were unremarkable. His laboratory values are shown in Table 1. Chest x-ray was negative for any acute pathology. EKG revealed normal sinus rhythm (NSR) at 85 beats per minute with isolated premature ventricular contractions (PVC) and a QTc of 423. 

**Table 1:** Initial laboratory values on admission.

Laboratory test	Value	Reference range
Hemoglobin	8.8 g/dL	13.2-16.6 g/dL
Iron	<10 mcg/dL	80-180 mcg/dL
Ferritin	10.9 ng/mL	12-300 ng/mL
Blood urea nitrogen	21 mg/dL	6-24 mg/dL
Creatinine	1.3 mg/dL	0.7-1.3 mg/dL
Total cholesterol	172 mg/dL	<200 mg/dL
Triglycerides	87 mg/dL	<150 mg/dL
High-density lipoprotein (HDL)	41 mg/dL	≥60 mg/dL
Low-density lipoprotein (LDL)	114 mg/dL	<100 mg/dL
Troponin I	<0.02ng/mL	0-0.04 ng/mL
Brain natriuretic peptide (BNP)	112 pg/mL	<100 pg/mL
Hemoglobin A1C	5.3%	<5.7%

On his first admission for a syncopal episode, a transthoracic echocardiogram (TTE) was performed which was significant for left ventricular (LV) ejection fraction (LVEF) of 40-45%. There was mild diffuse LV hypokinesis with mild tricuspid regurgitation (TR). He was diagnosed with heart failure with moderately reduced ejection fraction (HFmrEF) and was started on guideline-directed medical therapy and was followed up in an outpatient cardiology clinic.

**Figure 1:**
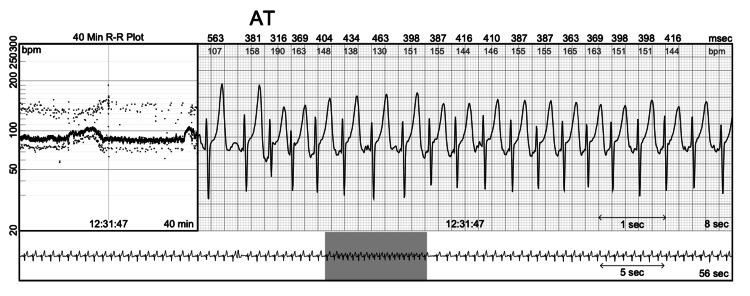
Recordings from Holter monitor showing AT. AT: atrial tachycardia

**Figure 2:**
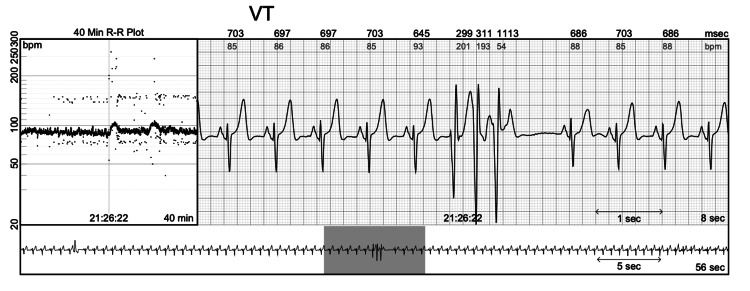
Recordings from Holter monitor showing VT. VT: ventricular tachycardia

During his outpatient visit, the patient was given a Holter monitor to rule out arrhythmias which revealed a predominant rhythm of NSR and 66 episodes of atrial tachycardia (AT), the longest being 20 beats at an average rate of 153 bpm (Figure 1). There were three episodes of ventricular tachycardia (VT), the longest being 15 beats at an average rate of 129 bpm up to 160 bpm (Figure 2). Premature atrial contraction (PAC) and premature ventricular contraction (PVC) burdens were 4.80% and 4.26%, respectively. 

Later, he underwent electrophysiology (EP) study for possible ablation of atrial tachycardia. The study revealed normal sinus node function and AV nodal conduction, decremental AV conduction, no dual AV nodal physiology, no AV nodal echoes, and a normal His-Purkinje system. There was decremental VA conduction, and no septal accessory pathway (with paraHisian pacing). No significant arrhythmia occurred spontaneously or with induction protocols during the study, with or without provocation. However, the patient was noted to have bouts of non-sustained ventricular tachycardia.

Subsequently, a dipyridamole pharmacologic stress test was ordered to rule out ischemic cardiomyopathy. The patient did not experience chest pain during stress; but there was maximal ~1 mm horizontal ST depression in leads V4-V6, II, III, and aVF during the recovery phase which suggested ischemia. Single-photon emission tomography imaging (SPECT) findings were remarkable for mild LV hypokinesis, more pronounced in the septum with borderline transient ischemic dilatation (TID). Large-sized, moderate degree reversible antero-apical defect extending to the septum and moderate size, moderate to severe degree partially reversible basal infero-septal defect. 

**Figure 3:**
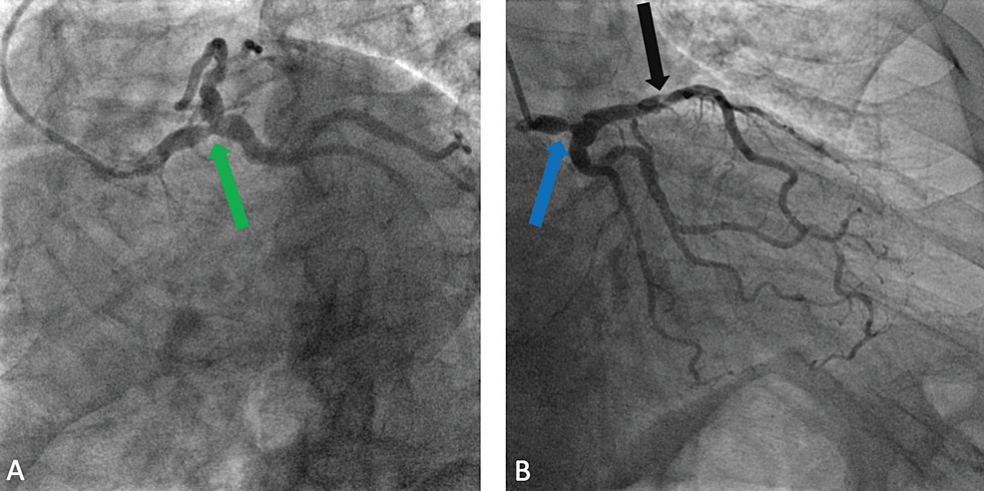
LAO caudal projection showing 80% distal LMCA stenosis (green arrow) (A). RAO caudal projection showing 80% distal LMCA stenosis (blue arrow) (B). There is a 40-45% mid-LAD artery stenosis (black arrow). LAO: left anterior oblique; LMCA: left main coronary artery; RAO: right anterior oblique; LAD artery: left anterior descending artery

**Figure 4:**
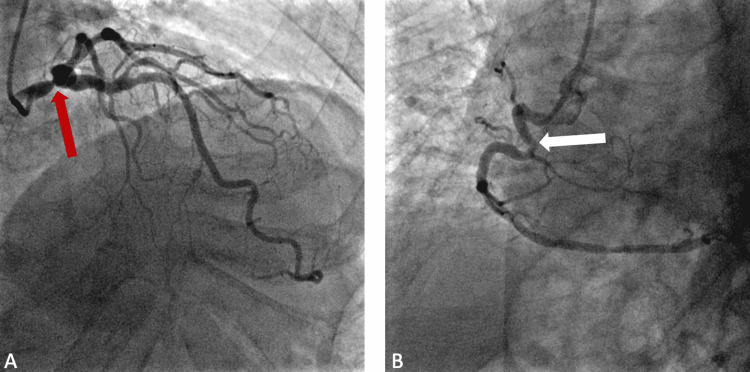
RAO cranial projection showing 80% distal LMCA stenosis (red arrow) (A). Proximal RCA 40% stenosis (white arrow) on a hairpin curve (B). RAO: right anterior oblique; LMCA: left main coronary artery; RCA: right coronary artery

Therefore, the patient was scheduled for a coronary angiogram. However, two days before the test, the patient presented to the emergency department with another pre-syncopal episode. The coronary angiogram was performed on this admission and was remarkable for severe left main disease. There was a discrete 80% stenosis in the distal third segment of the LMCA (Figures 3A, 3B, 4A). The lesion was without evidence of a thrombus. There was TIMI grade 3 flow through the vessel (brisk flow) and this was deemed as the culprit for the patient's clinical presentation. This was a bifurcation lesion. There was a discrete 50-70% stenosis in the ostial left anterior descending (LAD) coronary artery. The mid-LAD segment showed a 40-45% stenosis (Figure 3B). There was a discrete 70% stenosis in the proximal third segment of the second diagonal vessel. The proximal segment of the right coronary artery (RCA) showed a 40% stenosis on a hairpin curve (Figure 4B). LVEF calculated by contrast ventriculography was 45%. Given these findings, the patient was transferred for a prompt coronary artery bypass graft (CABG) surgery with subsequent resolution of further syncopal events on his follow-up visits.

## Discussion

Syncope is usually caused by cerebral hypoperfusion [7]. Differentials to consider during the workup of syncope, in this case, include vasovagal, orthostatic, drug-induced, arrhythmia, structural heart disease, and ischemic cardiomyopathy. This patient had recurrent self-limiting syncopal/presyncopal episodes with multiple hospital encounters. TTE was notable for reduced EF of 40-45% with mild diffuse LV hypokinesis. Holter monitor findings were significant for AT and VT. The subsequent EP study was unremarkable except for non-sustained bouts of ventricular tachycardia. However, the nuclear stress test showed borderline TID and a reversible anterior defect from the apex to the basal septum. A coronary angiogram revealed multi-vessel disease with a discrete 80% stenosis in the distal third segment of the LMCA which was the likely culprit for the patient's clinical presentation. The patient was promptly transferred to an outside facility with cardiothoracic surgery for CABG. 

Although rare, extensive transmural ischemia could lead to syncope. Kim and Bae presented a case of a 34-year-old male with significant LMCA stenosis and Brugada-type electrocardiography who initially presented with syncope [5]. Similarly, Li et al. presented a case of recurrent syncope in a 56-year-old male with EKG findings of multi-morphologic VT and coronary angiogram showing 90% stenosis of the LMCA [6]. In both cases, coronary revascularization with percutaneous coronary intervention (PCI) resulted in the resolution of symptoms at their one-year and three-month follow-up visits, respectively. 

In another study, Szymański et al. demonstrated that extensive transmural ischemia could lead to ventricular arrhythmias which is a known cause of syncope [8,9]. This explains the symptomatology in our patient with ventricular tachycardia in the setting of significant LMCA stenosis. Therefore, removing myocardial ischemia by coronary revascularization with CABG was deemed the best course of action. The resolution of further syncopal events post-surgical intervention upon a two-month clinic follow-up, further validates the etiology of the patient’s syncope. This rare presentation serves as an important reminder to consider ischemic heart disease in the evaluation of syncope.

## Conclusions

The workup of recurrent syncope entails a wide array of differentials. Here, we have presented a rare case of recurrent syncope attributed to malignant ventricular arrhythmia in the setting of LMCA stenosis. Our case is unique as we have presented a complete and comprehensive workup including EKG, echocardiogram, Holter monitor, EP study, and coronary angiogram. Eventually, resolution of symptoms was attained by correcting the myocardial ischemia with CABG. In this case report, we emphasize the importance of considering ischemic heart disease as a differential when there is a presentation of recurrent syncope.
